# Effect of a 21 day Daniel Fast on metabolic and cardiovascular disease risk factors in men and women

**DOI:** 10.1186/1476-511X-9-94

**Published:** 2010-09-03

**Authors:** Richard J Bloomer, Mohammad M Kabir, Robert E Canale, John F Trepanowski, Kate E Marshall, Tyler M Farney, Kelley G Hammond

**Affiliations:** 1 Cardiorespiratory/Metabolic Laboratory, The University of Memphis, Memphis, TN 38152, USA

## Abstract

**Background:**

Dietary modification via caloric restriction is associated with multiple effects related to improved metabolic and cardiovascular health. However, a mandated reduction in kilocalories is not well-tolerated by many individuals, limiting the long-term application of such a plan. The Daniel Fast is a widely utilized fast based on the Biblical book of Daniel. It involves a 21 day *ad libitum *food intake period, devoid of animal products and preservatives, and inclusive of fruits, vegetables, whole grains, legumes, nuts, and seeds. The purpose of the present study was to determine the efficacy of the Daniel Fast to improve markers of metabolic and cardiovascular disease risk.

**Methods:**

43 subjects (13 men; 30 women; 35 ± 1 yrs; range: 20-62 yrs) completed a 21 day period of modified food intake in accordance with detailed guidelines provided by investigators. All subjects purchased and prepared their own food. Following initial screening, subjects were given one week to prepare for the fast, after which time they reported to the lab for their pre-intervention assessment (day 1). After the 21 day fast, subjects reported to the lab for their post-intervention assessment (day 22). For both visits, subjects reported in a 12 hr fasted state, performing no strenuous physical activity during the preceding 24-48 hrs. At each visit, mental and physical health (SF-12 form), resting heart rate and blood pressure, and anthropometric variables were measured. Blood was collected for determination of complete blood count, metabolic panel, lipid panel, insulin, HOMA-IR, and C-reactive protein (CRP). Subjects' self-reported compliance, mood, and satiety in relation to the fast were also recorded. Diet records were maintained by all subjects during the 7 day period immediately prior to the fast (usual intake) and during the final 7 days of the fast.

**Results:**

Subjects' compliance to the fast was 98.7 ± 0.2% (mean ± SEM). Using a 10 point scale, subjects' mood and satiety were both 7.9 ± 0.2. The following variables were significantly (p < 0.05) lower following the fast as compared to before the fast: white blood cell count (5.68 ± 0.24 vs. 4.99 ± 0.19 10^3^·μL^-1^), blood urea nitrogen (13.07 ± 0.58 vs. 10.14 ± 0.59 mg·dL^-1^), blood urea nitrogen/creatinine (14.74 ± 0.59 vs. 11.67 ± 0.68), protein (6.95 ± 0.07 vs. 6.77 ± 0.06 g·dL^-1^), total cholesterol (171.07 ± 4.57 vs. 138.69 ± 4.39 mg·dL^-1^), LDL-C (98.38 ± 3.89 vs. 76.07 ± 3.53 mg·dL^-1^), HDL-C (55.65 ± 2.50 vs. 47.58 ± 2.19 mg·dL^-1^), SBP (114.65 ± 2.34 vs. 105.93 ± 2.12 mmHg), and DBP (72.23 ± 1.59 vs. 67.00 ± 1.43 mmHg). Insulin (4.42 ± 0.52 vs. 3.37 ± 0.35 μU·mL^-1^; p = 0.10), HOMA-IR (0.97 ± 0.13 vs.0.72 ± 0.08; p = 0.10), and CRP (3.15 ± 0.91 vs. 1.60 ± 0.42 mg·L^-1^; p = 0.13), were lowered to a clinically meaningful, albeit statistically insignificant extent. No significant difference was noted for any anthropometric variable (p > 0.05). As expected, multiple differences in dietary intake were noted (p < 0.05), including a reduction in total kilocalorie intake (2185 ± 94 vs. 1722 ± 85).

**Conclusion:**

A 21 day period of modified dietary intake in accordance with the Daniel Fast is 1) well-tolerated by men and women and 2) improves several risk factors for metabolic and cardiovascular disease. Larger scale, randomized studies, inclusive of a longer time period and possibly a slight modification in food choice in an attempt to maintain HDL cholesterol, are needed to extend these findings.

## Background

Caloric restriction has been reported in a number of studies to improve overall health in both humans [1] and animals [2]. Additionally, caloric restriction has been shown to increase the lifespan of many different species [3]. Although many hypotheses have been proposed in an attempt to explain these findings, the two that have garnered the greatest amount of credence in the scientific community are the oxidative damage attenuation hypothesis and the hormesis hypothesis. The oxidative damage attenuation hypothesis proposes that caloric restriction extends life by attenuating molecular oxidative damage [4]. The hormesis hypothesis poses that caloric restriction - through its function as a low-intensity stressor - builds up an animal's tolerance to higher-intensity stressors that would otherwise threaten its survival [5].

While the total amount of dietary energy appears important in regards to metabolic and cardiovascular health, the specific *type *of food consumed may be equally important [6,7]. This has been demonstrated in animal trials in which dietary modification (typically via protein manipulation or methionine manipulation) has been associated with improved health and longevity [8]. In human trials it has been noted that "natural" foods such as fruits, vegetables, whole grains, nuts and seeds, which contain little or no preservatives, little to no saturated fats, with an abundance of fiber and micronutrients, may provide health enhancing properties [9-11]. Such a diet is often lower in total kilocalories by default, as these foods provide greater satiety than more energy-dense foods [12]. It follows that a dietary approach inclusive of these food choices may be considered ideal for individuals seeking enhanced health through nutrient intake.

Although many diet plans are currently available, those used in research studies focused on caloric restriction for improved health and longevity routinely involve a reduction of dietary energy equal to approximately 20 - 40% of an individual's usual intake [13]. Other plans involve short periods of caloric restriction followed by normal intake, done in a cyclic pattern [14]. Other plans involve "alternate day" fasting, in which no food is consumed for one day, followed by *ad libitum *intake on the subsequent day, done in a cyclic pattern [15]. The one commonality of these diet plans is that individuals can maintain their usual food choices; they simply need to eat less of those foods. This regimen assumes that the *quantity *of food is more important than the *quality*.

An alternative approach that has received a great deal of attention in recent years from the non-scientific community is the "Daniel Fast". The concept of the Daniel Fast comes from Daniel 1:8-14 (NIV), "But Daniel resolved not to defile himself with the royal food and wine, and he asked the chief official for permission not to defile himself this way. Now God had caused the official to show favor and sympathy to Daniel, but the official told Daniel, 'I am afraid of my lord the king, who has assigned your food and drink. Why should he see you looking worse than the other young men your age? The king would then have my head because of you.' Daniel then said to the guard whom the chief official had appointed over Daniel, Hananiah, Mishael and Azariah, 'Please test your servants for ten days: Give us nothing but vegetables to eat and water to drink. Then compare our appearance with that of the young men who eat the royal food, and treat your servants in accordance with what you see.' So he agreed to this and tested them for ten days." Other Biblical translations replace the word "vegetables" with "pulse", which indicates "food grown from seed".

An additional reference to this fast is found in Daniel 10:2-3 (NIV): "At that time I, Daniel, mourned for three weeks. I ate no choice food; no meat or wine touched my lips; and I used no lotions at all until the three weeks were over." Based on this latter passage, a Daniel Fast is most commonly partaken for 21 days, although fasts of 10 and 40 days have also been observed. A Daniel Fast involves *ad libitum *intake of specific foods, but the food choices are restricted to essentially fruits, vegetables, whole grains, nuts, seeds, and oil. This plan resembles a vegan diet, which has been reported to yield health enhancing properties [16,17]. However, a Daniel Fast is more stringent, in that aside from the exclusion of all animal products, there are no processed foods, white flour products, preservatives, additives, sweeteners, flavorings, caffeine, or alcohol allowed in this plan. Despite this, as individuals traditionally follow this fast for religious purposes in an attempt to become "closer to God" during a time of extended prayer, anecdotal reports indicate that maintenance of the fast can be achieved by most.

The aim of the present study was to investigate the metabolic and cardiovascular health effects of the Daniel Fast in human subjects. To our knowledge, this was the first scientific investigation of the Daniel Fast. As such, this study simply involved a pre/post assessment and did not include random assignment to two different dietary plans. Moreover, we did not attempt to limit recruitment only to those with known risk factors for cardiovascular and metabolic disease, as we were curious to see how all individuals responded to such a dietary plan. In addition to our interest in the measured anthropometric, hemodynamic, and biochemical outcome variables, our objective with this initial study was to determine the feasibility of maintaining this dietary lifestyle, as well as to determine how subjects complied with and tolerated the fast.

## Methods

### Subjects and Screening

Forty-four subjects (13 men; 31 women) were initially recruited to participate and were enrolled in this study. The mean age of subjects was 35 ± 1 years, with a range of 20-62 years. One female subject had a diagnosis of well-controlled type II diabetes (and used oral hypoglycemic agents), and one male subject had a history of coronary artery bypass graft surgery (and used both a statin and Plavix^®^). Three subjects were hypertensive upon enrollment (BP≥140/90 mmHg; 2 men and 1 woman) and seven had hypercholesterolemia (total cholesterol > 200 mg·dL^-1^; 1 man and 6 women). One man used a beta blocker and one man used an anti-depressant. Three women used anti-depressants, six used oral contraceptives, two used estrogen replacement, two used a sleep aid, one used a statin, and one used an angiotensin II receptor antagonist.

Six female subjects were vegetarian prior to starting the fast. No restrictions were placed on subjects regarding body mass necessary for enrollment. Hence, the BMI of subjects ranged from 18.0 kg·m^-2 ^to 40.6 kg·m^-2^, with 21 subjects classified as normal weight (BMI < 25 kg·m^-2^), 10 classified as overweight (BMI 25-29.9 kg·m^-2^), and 13 classified as obese (BMI ≥30 kg·m^-2^). All subjects were nonsmokers. Of the 44 subjects, 34 were classified as exercise trained, performing 1.8 ± 0.24 hours of anaerobic and 3.0 ± 0.23 hours of aerobic exercise per week for the past 5.4 ± 0.82 and 7.7 ± 1.0 years, respectively. Subjects were classified as exercise trained if they regularly performed a combined minimum of three hours per week of anaerobic and aerobic exercise of moderate to high intensity. Collectively, subjects were relatively healthy, active men and women. Eligibility and classification was determined by completion of health history, drug and dietary supplement usage, and physical activity questionnaires. Prior to participation, each subject was informed of all procedures, potential risks, and benefits associated with the study through both verbal and written form in accordance with the approved procedures of the University Institutional Review Board for Human Subjects Research (H10-06). Subjects signed an informed consent form prior to being admitted as a subject.

During the initial visit to the laboratory, subjects completed all paperwork, were provided detailed instructions for the fast, were given food logs for dietary recording and reviewed food models in an attempt to improve accuracy of recording (as described below), and were provided a calendar outlining their full participation. While numerous websites are available with information and recipes related to the Daniel Fast, subjects were provided a detailed outline of those foods that are allowed, as well as commonly consumed foods that are not allowed. A recipe guide was also provided. It is important to note that subjects purchased and prepared their own food. Subjects returned to the lab 1-2 weeks later to have baseline assessments performed and to begin the 21 day fast. The outcome variables described below were measured before (baseline: day 1 of the fast) and after the fast (day 22). All data collection was done in the morning hours (5:00-11:00 am) in a 12 hour fasted state.

### SF-12 Questionnaire

Upon arrival to the lab, subjects were asked to complete a questionnaire pertaining to their overall mental and physical health status (SF-12v2; QualityMetric, Inc.). The questionnaire was delivered using a computer based program and scoring was performed using automated software immediately following completion of the questionnaire.

### Heart Rate and Blood Pressure

Following completion of the above questionnaire, subjects were asked to void. Women performed a urine pregnancy test to confirm that they were not pregnant, as pregnant women were not allowed to participate in this study due to potential fetal radiation exposure with the dual energy x-ray absorptiometry (DEXA) scan. Subjects were then seated in a chair with a blood pressure cuff placed on their left arm. Subjects rested for 10 minutes, after which time heart rate was measured via palpation for 60 seconds using the radial artery (by two trained technicians--one on each wrist). Blood pressure was then measured via auscultation using a calibrated manometer and a dual earpiece stethoscope that allowed for two trained technicians to listen simultaneously. Duplicate measures were obtained for both heart rate and blood pressure (hence a total of four measures for each variable, two from each technician) and the average of all measures was used in data analysis. If values deviated by more than 5 bpm for heart rate or 5 mmHG for blood pressure, an additional measure was taken.

### Anthropometric Variables

Subjects' height was measured using a stadiometer, and body weight was measured using a calibrated medical scale. Body mass index was calculated as weight (kg) divided by height (m^2^). Waist and hip circumference measurements were obtained using a tension-regulated tape measure, with subjects wearing "spandex-like" shorts. Body composition was determined by DEXA (Hologic QDR-4500W) using a 4-minute fan array. Specifically, total and regional (trunk specific) body fat were determined. The assessment was performed by a licensed technician.

### Blood Collection and Biochemical Variables

Venous blood samples were taken from subjects' forearm via needle and Vacutainer™ by a trained phlebotomist. Following collection, samples were processed accordingly to obtain plasma/serum. All assays were performed with 24 hours of sample collection. Complete blood count was determined using an automated cell counter (Coulter LH750). The comprehensive metabolic panel was determined using automated procedures (Roche/Hitachi Modular). The lipid panel was determined using enzymatic procedures (Roche/Hitachi Modular). Insulin was determined using an immuno-chemiluminescent assay procedure (Roche Modular E170). C-reactive protein was determined using a high-sensitivity, particle-enhanced turbidimetric immunoassay (Roche Integra 800). The homeostasis model assessment (HOMA-IR) was used as an index of insulin resistance [18] and calculated as: [fasting glucose (mg·dL^-1^) × fasting insulin (μU·mL^-1^)]/405.

### Dietary Records and Physical Activity

All subjects were instructed to maintain their normal diet until they began the fast and to record on data forms all food and beverage consumed during the seven days immediately prior to the start of the fast. Subjects were also asked to record food and beverage intake during the final seven days of the fast. A technician reviewed in detail the nutritional records with each subject upon receipt. Records were analyzed by using Food Processor SQL, version 9.9 (ESHA Research, Salem, OR). Regarding physical activity, subjects were instructed to maintain their normal habits during the entire study period. Subjects were instructed to refrain from alcohol consumption during the fast and to avoid strenuous exercise during the 24-48 hours immediately preceding the two assessment days.

### Compliance, Mood, and Satiety

On a scale of 0-100 (0 = complete non-compliance, 100 = complete compliance), subjects rated their overall compliance to the fast, with regards to food choices. Using a 10 point visual analog scale (0 = worst possible mood/extreme hunger, 10 = best possible mood/complete satiety), subjects rated their overall mood and feeling of satiety while on the fast.

### Statistical Analysis

All data were analyzed using a t-test. Analyses were performed using JMP statistical software (version 4.0.3, SAS Institute, Cary, NC). Statistical significance was set at P ≤ 0.05. The data are presented as mean ± SEM.

## Results

### Compliance, Mood, and Satiety

Forty-four subjects were initially enrolled in the study, and all 44 subjects completed the 21 day fast, as well as pre and post assessments. However, one subject reported a compliance rate of only 60% to the fast and was therefore excluded from data analysis. Of the remaining 43 subjects, compliance to the fast was 98.7 ± 0.2%. Subjects' overall mood during the fast was indicated at 7.9 ± 0.2, while satiety during the fast was indicated at 7.9 ± 0.2.

Through completion of a post fast questionnaire, subjects reported that the main enervation of their mental health was the omission of caffeine. However, aside from caffeine, there was no one particular food item that impaired subjects' mental outlook or limited subjects' interest in complying with the fast guidelines. Subjects noted that they enjoyed the *ad libitum *nature of the plan, as well as the wide variety of food choices. In fact, most subjects reported that they would continue implementing many components of the plan into their previous diets. Finally, subjects reported that selecting food items that complied with the fast guidelines, in addition to planning and preparing meals, were the most challenging components of the fast. That being said, subjects commented repeatedly that the forced review of food labels significantly increased their knowledge and awareness of the kilocalorie content and macro- and micro-nutrient composition of foods, as well as the inclusion of additives and preservatives. Hence, such a program may very well serve as an education in food choices, rather than merely a method to improve metabolic and cardiovascular health.

In relation to the bloodborne variables, we were able to obtain blood samples on 42 of the 43 subjects. Of the 42 subjects for which blood samples were obtained, pre and post insulin values were not included for two subjects and CRP values were not included for one subject, due to problems in sample analysis.

### Mental and Physical Health, Hemodynamic, and Anthropometric Data

No differences were noted from pre to post fast for mental (p = 0.45) or physical (p = 0.93) health. No differences were noted from pre to post fast for body weight (p = 0.52), BMI (p = 0.43), waist circumference (p = 0.65), hip circumference (p = 0.62), waist:hip (p = 0.86), percent total body fat (p = 0.86), % trunk body fat (p = 0.77), fat mass (p = 0.64), or fat free mass (p = 0.60). No difference was noted from pre to post fast for resting heart rate (p = 0.13). However, systolic (p = 0.007) and diastolic (p = 0.03) blood pressure were both lower post fast compared to pre fast. Data for all variables are presented in Table 1.

**Table 1:** Mental and physical health, hemodynamic, and anthropometric data of men and women before and after a 21 day Daniel Fast

Variable	Pre	Post
Mental Health	53.5.6 ± 0.9	51.7 ± 1.2
Physical Health	55.6 ± 0.6	55.6 ± 0.7
Heart Rate (bpm)	68.2 ± 1.7	64.5 ± 1.4
Systolic Blood Pressure (mmHg)*	114.7 ± 2.3	105.9 ± 2.1
Diastolic Blood Pressure (mmHg)**	72.2 ± 1.6	67.0 ± 1.4
Weight (kg)	77.5 ± 3.0	74.7 ± 2.7
BMI (kg·m^-2^)	27.0 ± 0.9	26.0 ± 0.8
Waist (cm)	92.2 ± 2.0	90.4 ± 2.0
Hip (cm)	105.8 ± 1.8	104.5 ± 1.7
Waist:Hip	0.87 ± 0.01	0.87 ± 0.01
Total Body Fat (%)	30.2 ± 1.6	29.9 ± 1.6
Trunk Body Fat (%)	29.7 ± 1.7	29.1 ± 1.7
Fat Mass (kg)	24.2 ± 1.9	23.1 ± 1.8
Fat Free Mass (kg)	53.8 ± 2.0	52.1 ± 1.9

Values are mean ± SEM.

* p = 0.007

** p = 0.03

No other statistically significant differences noted (p > 0.05).

### Biochemical Data

With the exception of white blood cells, which were lower post fast compared to pre fast (p = 0.03), no differences were noted for complete blood count variables (p > 0.05; Table 2). With the exception of blood urea nitrogen (p = 0.003), blood urea nitrogen:creatinine (p = 0.005), and protein (p = 0.05), which were all lower post fast compared to pre fast, no differences were noted for metabolic panel variables (p > 0.05; Table 3). Total cholesterol (p < 0.0001), LDL-C (p = 0.0004), and HDL-C (p = 0.02) were all lower post fast compared to pre fast. No difference was noted for VLDL (p = 0.12), Total:HDL (p = 0.47), or triglyceride (p = 0.12). Data are presented in Table 4. Insulin (pre: 4.42 ± 0.52 vs. post: 3.37 ± 0.35 μU·mL^-1^; p = 0.10), HOMA-IR (pre: 0.97 ± 0.13 vs. post: 0.72 ± 0.08; p = 0.10), and CRP (pre: 3.15 ± 0.91 vs. post: 1.60 ± 0.42 mg·L^-1^; p = 0.13) were lowered in a clinically meaningful manner, although this decline failed to reach statistical significance.

**Table 2:** Complete blood count data of men and women before and after a 21 day Daniel Fast

Variable	Pre	Post
WBC (10^3^·μL^-1^)*	5.7 ± 0.2	5.0 ± 0.2
RBC (10^6^·μL^-1^)	4.4 ± 0.1	4.4 ± 0.1
Hemoglobin (g·dL^-1^)	13.7 ± 0.2	13.7 ± 0.2
Hematocrit (%)	39.7 ± 0.5	39.6 ± 0.5
MCV (fL)	89.3 ± 0.6	89.5 ± 0.6
MCH (pg)	30.9 ± 0.3	31.0 ± 0.3
MCHC (g·dL^-1^)	34.6 ± 0.1	34.6 ± 0.1
RDW (%)	13.3 ± 0.2	13.2 ± 0.2
Platelets (10^3^·μL^-1^)	237.5 ± 8.9	222.5 ± 8.5
Neutrophils (%)	55.8 ± 1.4	54.1 ± 1.7
Lymphocytes (%)	33.9 ± 1.2	35.2 ± 1.5
Monocytes (%)	7.0 ± 0.3	7.7 ± 0.3
Eosinophils (%)	2.8 ± 0.3	2.4 ± 0.2
Basophils (%)	0.5 ± 0.1	0.6 ± 0.1

Values are mean ± SEM.

* p = 0.03

No other statistically significant differences noted (p > 0.05).

**Table 3:** Metabolic panel data of men and women before and after a 21 day Daniel Fast

Variable	Pre	Post
Glucose (mg·dL^-1^)	86.9 ± 1.5	84.8 ± 1.0
BUN (mg·dL^-1^)*	13.1 ± 0.6	10.1 ± 0.6
Creatinine (mg·dL^-1^)	0.9 ± 0.0	0.9 ± 0.0
BUN:Creatinine**	14.7 ± 0.6	11.7 ± 0.7
Sodium (mmol·L^-1^)	138.7 ± 0.3	139.1 ± 0.3
Potassium (mmol·L^-1^)	4.3 ± 0.1	4.4 ± 0.1
Chloride (mmol·L^-1^)	102.6 ± 0.3	102.6 ± 0.3
CO_2 _(mmol·L^-1^)	25.3 ± 0.3	25.9 ± 0.3
Calcium (mg·dL^-1^)	9.4 ± 0.1	9.5 ± 0.1
Protein (g·dL^-1^)***	7.0 ± 0.1	6.8 ± 0.1
Albumin (g·dL^-1^)	4.3 ± 0.0	4.2 ± 0.0
Globulin (g·dL^-1^)	2.7 ± 0.1	2.6 ± 0.1
A:G	1.6 ± 0.0	1.6 ± 0.0
Bilirubin (mg·dL^-1^)	0.5 ± 0.0	0.6 ± 0.1
Alk Phos (IU·L^-1^)	63.8 ± 3.3	64.7 ± 3.1
AST (SGOT) (IU·L^-1^)	23.0 ± 1.6	21.4 ± 0.6
ALT (SGPT) (IU·L^-1^)	19.7 ± 1.2	18.0 ± 0.9

Values are mean ± SEM.

* p = 0.003

** p = 0.005

*** p = 0.05

No other statistically significant differences noted (p > 0.05).

**Table 4:** Lipid panel data of men and women before and after a 21 day Daniel Fast

Variable	Pre	Post
Cholesterol (mg·dL^-1^)*	171.1 ± 4.6	138.7 ± 4.4
Triglycerides (mg·dL^-1^)	85.1 ± 4.8	75.3 ± 3.6
HDL-C (mg·dL^-1^)**	55.6 ± 2.5	47.6 ± 2.2
VLDL-C (mg·dL^-1^)	17.0 ± 1.0	15.0 ± 0.7
LDL-C (mg·dL^-1^)***	98.4 ± 3.9	76.1 ± 3.5
Total:HDL-C	3.3 ± 0.1	3.1 ± 0.1

Values are mean ± SEM.

* p < 0.0001

** p = 0.02

*** p = 0.0004

No other statistically significant differences noted (p > 0.05).

### Dietary Data

As expected, several differences existed in dietary intake from pre fast to the final week of the fast. These included a decrease in total kilocalories (p = 0.0005), protein grams (p = 0.0003), the percent of protein (p = 0.004), fat grams (p = 0.003), saturated fat (p < 0.0001), trans fat (p = 0.006), and cholesterol (p < 0.0001). An increase in the percent of carbohydrate intake (p = 0.0002), fiber (p < 0.0001), and vitamin C (p = 0.002) was also noted. Data are presented in Table 5.

**Table 5:** Dietary data of men and women before and during the final seven days of a 21 day Daniel Fast

Variable	Pre	During	P value
Kilocalories	2185 ± 94	1722 ± 85	0.0005
Protein (g)	92 ± 6	62 ± 5	0.0003
Protein (%)	17 ± 0	13 ± 0	0.004
Carbohydrate (g)	287 ± 14	269 ± 17	0.41
Carbohydrate (%)	53 ± 0	62 ± 0	0.0002
Fiber (g)	26 ± 2	40 ± 3	< 0.0001
Sugar (g)	95 ± 7	86 ± 6	0.37
Fat (g)	74 ± 5	54 ± 4	0.003
Fat (%)	30 ± 0	27 ± 0	0.20
Saturated Fat (g)	24 ± 2	9 ± 1	< 0.0001
Monounsaturated Fat (g)	14 ± 2	14 ± 2	0.89
Polyunsaturated Fat (g)	8 ± 1	9 ± 1	0.47
Trans Fat (g)	1 ± 0	0 ± 0	0.006
Omega 3 (mg)	711 ± 163	798 ± 202	0.77
Omega 6 (mg)	2510 ± 327	3341 ± 345	0.10
Cholesterol (mg)	225 ± 19	28 ± 20	< 0.0001
Vitamin C (mg)	70 ± 9	119 ± 12	0.002
Vitamin E (mg)	8 ± 2	11 ± 1	0.36
Vitamin A (RE)	404 ± 60	435 ± 70	0.70

Values are mean ± SEM.

## Discussion

Results from the present study indicate that a 21 day Daniel Fast 1) significantly reduces systolic and diastolic blood pressure, 2) significantly reduces total, LDL, and HDL cholesterol, 3) reduces insulin, HOMA-IR, and C-reactive protein in a clinically meaningful, although statistically insignificant manner, 4) does not cause any negative effects on blood count or metabolic panel values, 5) is well-tolerated, and 6) may be useful as a nutrition education tool for men and women. To our knowledge, this is the first scientific investigation of the Daniel Fast. Subsequent statistical analyses indicated no interactions between normal weight and overweight/obese subjects, men and women, and exercise trained and untrained subjects (as discussed below). This suggests that a wide variety of individuals may benefit from a dietary approach in accordance with the Daniel Fast. As we were not concerned with weight status, sex, or training status comparisons in this initial study, coupled with the fact that no interactions were noted for any of the above-mentioned comparisons, only pooled data are presented in the tables and discussed within this manuscript.

It is important to note that our findings are in reference to relatively healthy, young to middle age men and women (age range: 20-62 years), with a wide BMI range (18.0 kg·m^-2 ^to 40.6 kg·m^-2^), and varied regular exercise and dietary habits. Interestingly, we noted similar findings in all subject groups (as discussed below). For example, similar *percent changes *in all measured variables from pre to post fast were observed in subjects who are of normal weight and low body fat (e.g., < 10% for men and < 20% for women), who exercise regularly for 4+ hours per week and eat a very "clean" diet on a regular basis (including vegetarians), as compared to overweight/obese, sedentary subjects who consume a relatively poor diet. However, it should be noted that while the percent change in outcome measures was similar between such individuals, the absolute values were better for the normal weight, exercise enthusiasts compared to the overweight/obese, sedentary subjects (discussed below). Based on our collective findings, it is possible that individuals with diagnosed metabolic and cardiovascular disorders may experience clinically meaningful results on such a dietary regimen. Future work should consider the inclusion of such patients, as this diet may be considered an anti-inflammatory, anti-atherogenic, anti-hypertensive, non-pharmacologic approach to disease risk management.

While we did note a reduction of blood pressure, as well as a decrease in total (19%) and LDL cholesterol (23%) with the Daniel Fast in just three weeks, findings similar to those noted for other plant-based diets [19-21], we also noted a decrease in HDL-C (14%). While the total:HDL-C was improved slightly (Table 4), the drop in HDL-C remains a concern. If this eating plan is to be viewed as "heart healthy" in all aspects, future studies involving the Daniel Fast may include specific food choices (e.g., almonds, *plantago ovata *husks, walnuts) [22-24] and/or dietary supplements [25,26] noted to increase HDL-C, in an effort to maintain HDL-C while decreasing both total and LDL-C. Aside from HDL-C, although insulin (24%), HOMA-IR (26%), and C-reactive protein (49%) decreased during the 21 day fast, these decreases failed to reach statistical significance. A *post hoc *power analysis indicated that a total of 57 subjects would be needed to demonstrate statistical significance for insulin and HOMA-IR, while 65 subjects would be needed for C-reactive protein. Future studies may include a larger sample size in order to improve the chance of achieving statistical significance for these variables. Of course, inclusion of individuals with high pre fast values for these variables may also improve the chance of noting statistically significant findings.

It is interesting to note that even those subjects who were vegetarian prior to starting the fast experienced dramatic reductions in total and LDL-C, in addition to improvements in other markers. Clearly, the exclusion of meat from the diet (as is the case for vegetarians) is not the only dietary factor involved in raising circulating cholesterol and other risk factors for cardiovascular and metabolic disease. It is possible that multiple dietary factors, inclusive moderate and high fat dairy products, as well as processed and packaged foods containing trans fats, simple carbohydrates, and various additives and preservatives can negatively affect the variables included in the present design. Future study is needed to investigate this effect.

Aside from the previously discussed variables, significant effects were noted for white blood cells (Table 2), blood urea nitrogen, blood urea nitrogen:creatinine, and protein (Table 3). While the latter findings are likely related to the lower protein intake (particularly meat) observed in this study [27,28], the slight decrease in white blood cells is not well-supported. It has been suggested that ingestion of food additives and preservatives can increase white blood cell count by triggering an immune response due to a sensing of invading pathogens from the food stuff; however, we are unaware of any scientific reports that confirm this hypothesis. In the present plan, subjects' diets were devoid of such additives and preservatives, which may help to explain the finding of lowered white blood cells. Moreover, any reduction in saturated fat intake, as well as in body weight or body fat levels, may have contributed to these findings, as excess fat ingestion and increased adiposity has been associated with increased inflammation.

Finally, in relation to our measured variables, although we noted some positive changes in body weight and other anthropometric variables from pre to post fast, no change was of statistical significance (Table 1). This indicates that the noted changes of significance in terms of the bloodborne variables were not dependent on simple changes in body weight/fat. While both the type and quality of food was indeed different while on the fast compared to pre fast, the total kilocalorie intake of subjects was also reduced approximately 20% (Table 5), despite the freedom of *ad libitum *intake. This finding indicates that the intake of natural, high fiber, low glycemic carbohydrate foods enhances satiety and consequently reduces kilocalorie intake [12,29]. Subjects did not purposely restrict food intake, as evidenced by their comments, compliance to the fast, and self-reported satiety ratings. It is likely that subjects consumed a similar or greater *volume *of food as compared to pre fast, despite consuming fewer total kilocalories; this is plausible, because the foods that were consumed during the fast were more nutrient dense and less energy dense compared to the foods consumed before the fast. This includes the increased intake of fruit during the fast, which resulted in a similar amount of sugar to be consumed as compared to pre fast, although seemed to provide increased satiety, likely due to the fiber content of some fruits. Despite this, from a pure weight loss point of view, it is likely that a longer fast duration (> 21 days) would be necessary to observe statistically significant and clinically meaningful changes in anthropometric variables in our subjects. As mentioned earlier, many of our subjects were already in very good physical condition prior to starting the fast, and therefore had little room for improvement. Inclusion of a homogeneous sample of obese subjects would likely yield statistically significant findings for many of the anthropometric variables.

Besides the anthropometric variables, we noted no changes of statistical significance with regards to the blood count (Table 2), metabolic panel (Table 3), or lipid panel (Table 4) variables not previously discussed. In fact, most variables were nearly identical from pre to post fast. Based on these findings, the short-term effects of the Daniel Fast do not pose any health concerns. Future studies including longer fasting protocols should continue to monitor clinical parameters in order to extend the present findings.

Although our intention with this study was not to make comparisons between normal weight and overweight/obese subjects, in an attempt to clarify our findings we also analyzed data using a 2 (weight status: normal weight vs. overweight/obese) × 2 (pre/post fast) analysis of variance. No interaction effects were noted for any variable (p > 0.05), indicating that normal weight and overweight/obese subjects respond to the Daniel Fast in a similar manner. However, aside from having higher body weight, BMI, total and trunk percent body fat and fat mass, and waist:hip (p < 0.0001), overweight/obese subjects were noted as having higher heart rate (p = 0.01), systolic blood pressure (p = 0.0001), diastolic blood pressure (p = 0.005), glucose (p = 0.007), insulin (p < 0.0001), HOMA-IR (p = 0.002), total cholesterol (p = 0.003), triglycerides (p = 0.02), VLDL-C = 0.03), LDL-C (p < 0.0001), and total:HDL-C (p < 0.0001) as compared to normal weight subjects. Each of these differences was expected based on prior literature.

We also analyzed data using a 2 (sex) × 2 (pre/post fast) analysis of variance. No interaction effects were noted for any variable (p > 0.05), indicating that men and women respond to the Daniel Fast in a similar manner. Men were noted as having higher body weight (p < 0.0001), fat free mass (p < 0.0001), waist:hip (p < 0.0001), systolic blood pressure (p = 0.0002), diastolic blood pressure(p < 0.0001), glucose (p < 0.0001), insulin (p = 0.008), HOMA-IR (p = 0.002), blood urea nitrogen (p = 0.006), and total:HDL-C (p = 0.04) as compared to women. Women were noted as having higher total percent body fat (p < 0.0001), total cholesterol (p = 0.02), and HDL-C (p = 0.0007) compared to men.

Finally, for completeness of analysis, we analyzed data using a 2 (training status: trained vs. untrained) × 2 (pre/post fast) analysis of variance. No interaction effects were noted for any variable (p > 0.05), indicating that exercise trained and untrained men and women respond to the Daniel Fast in a similar manner. Untrained subjects were noted as having higher BMI (p = 0.04), total (p = 0.0003) and trunk (p = 0.002) percent body fat, and fat mass (p = 0.02), as well as total cholesterol (p = 0.006) and LDL-C (p = 0.05) as compared to trained subjects. Trained subjects had higher fat free mass (p = 0.03) as compared to untrained subjects.

While it is obvious that the change in subjects' dietary intake is responsible for our findings, difficulty lies in determining which specific dietary component is most important in this regard. Because we have included multiple outcome measures in this initial study, in addition to the measurement of multiple dietary variables, a complete analysis of predictor and response variables would be the topic of another manuscript. It is likely that the combination of decreased kilocalorie, saturated fat, and cholesterol intake, coupled with an increase in nutrient and fiber-rich fruit, vegetable, and whole grain intake, contributed to our findings. Moreover, it is possible that the elimination of food additives, preservatives, and processing agents, in addition to the decrease in protein intake (methionine included), could be partly responsible for our findings. Future study is needed, ideally inclusive of a larger sample of subjects, to provide more definitive answers to the above hypotheses.

## Conclusion

Our data indicate that exercise trained and untrained individuals, inclusive of normal weight, overweight, and obese men and women can benefit from a dietary approach in accordance with the Daniel Fast. This is evidenced by reductions in total and LDL cholesterol, systolic and diastolic blood pressure, and trends for reductions in insulin, HOMA-IR, and C-reactive protein. The diet plan was well-tolerated by subjects, with several noting a desire to continue with the plan long-term, and appears to provide a default nutrition education. Follow-up studies should include randomized designs, possibly inclusive of small daily allotments of dairy and lean meat/fish in order to boost compliance and to minimize long-term nutrient deficiencies. Such studies would seek to determine the feasibility of both a standard and slightly modified Daniel Fast for long-term intake, as well as modifications of this approach for maintenance within a lifestyle eating plan aimed at optimal health, inclusive of food choices and/or nutritional supplements aimed at maintaining HDL-C. In particular, such studies may include patients with diagnosed metabolic (e.g., pre-diabetes and diabetes) and cardiovascular disorders (e.g., hypertension and hypercholesterolemia), as such individuals may experience clinically relevant results manifesting in improved disease prognosis.

## Competing interests

The authors declare that they have no competing interests.

## Authors' contributions

RJB was responsible for the study design, oversight and analysis of biochemical variables, statistical analyses, and writing of the manuscript. MMK was responsible for coordination of the study. MMK, REC, JFT, KEM, and TMF were responsible for subject recruitment, screening, and retention, data collection and entry, and blood collection and processing. KGH was responsible for performing the DEXA scans. All authors assisted in reviewing/editing the manuscript and all authors reviewed and approved of the final manuscript.
